# Resolution of Mild Ganciclovir-Resistant Cytomegalovirus Disease with Reduced-Dose Cidofovir and CMV-Hyperimmune Globulin

**DOI:** 10.1155/2014/342319

**Published:** 2014-06-01

**Authors:** Samir J. Patel, Samantha A. Kuten, Richard J. Knight, Dana M. Hong, A. Osama Gaber

**Affiliations:** ^1^Department of Pharmacy, Houston Methodist Hospital, 6565 Fannin Street, DB1-09, Houston, TX 77030, USA; ^2^Department of Surgery, Houston Methodist Hospital, 6550 Fannin Street, SM 1661A, Houston, TX 77030, USA; ^3^J. C. Walter Jr. Transplant Center, Houston Methodist Hospital, 6550 Fannin Street, SM 1201, Houston, TX 77030, USA

## Abstract

Ganciclovir-resistant cytomegalovirus (CMV) is associated with significant morbidity in solid organ transplant recipients. Management of ganciclovir-resistant CMV may be complicated by nephrotoxicity which is commonly observed with recommended therapies and/or rejection induced by “indirect” viral effects or reduction of immunosuppression. Herein, we report a series of four high serologic risk (donor CMV positive/recipient CMV negative) kidney transplant patients diagnosed with ganciclovir-resistant CMV disease. All patients initially developed “breakthrough” viremia while still receiving valganciclovir prophylaxis after transplant and were later confirmed to exhibit UL97 mutations after failing to eradicate virus on adequate dosages of valganciclovir. The patients were subsequently and successfully treated with reduced-dose (1-2 mg/kg) cidofovir and CMV-hyperimmune globulin, given in 2-week intervals. In addition, all patients exhibited stable renal function after completion of therapy, and none experienced acute rejection. The combination of reduced-dose cidofovir and CMV-hyperimmune globulin appeared to be a safe and effective regimen in patients with mild disease due to ganciclovir-resistant CMV.

## 1. Introduction

Cytomegalovirus (CMV) is a significant cause of morbidity among solid organ transplant recipients. Active infection can result in the well-defined direct effects of either CMV syndrome or tissue-invasive disease, while the “indirect effects” include potential immune-mediated injury of the allograft as well as an increased propensity for coinfections [1]. Ganciclovir and its prodrug, valganciclovir, each have been effective in both prevention and treatment of CMV disease [2–4]. However, the emergence of ganciclovir-resistant (GCV-R) CMV has posed a more significant threat due to an aggressive disease course and a greater mortality risk [5].

Treatment options for GCV-R CMV are limited, with foscarnet being recommended as the initial treatment option followed by cidofovir [6]. Although these agents are known to have activity against CMV, both are associated with substantial side effects, the most notable of which is nephrotoxicity. Significant renal injury with these agents has been reported in 30–60% of patients and may even occur after only 1-2 doses [7, 8]. Furthermore, cidofovir is contraindicated in patients with an estimated creatinine clearance of <55 mL/min or a serum creatinine of >1.5 mg/dL [8], which are common findings among the solid organ transplant population.

Herein, we report a successful strategy of reduced-dose cidofovir in combination with CMV-hyperimmune globulin (CMV-IgG) in four consecutive kidney transplant recipients with varying degrees of renal impairment and mild cases of genotypically confirmed GCV-R CMV. We chose to use cidofovir instead of foscarnet in these cases based on our own disappointing experience with the latter agent, having observed a high rate of acute renal failure requiring dialysis, in patients with severe GCV-R CMV disease. The rationale for reduced-dose cidofovir was the impaired baseline renal function observed in our patients as well as the ability to use probenecid and hydration as nephroprotective measures. CMV-IgG was provided as an adjunct to antiviral therapy, as well as for its potential immunomodulatory properties. Use of this regimen resulted in viral clearance, preservation of graft function, and avoidance of rejection in all four cases.

## 2. Methods

Four consecutive kidney transplant recipients with documented ganciclovir-resistant CMV were included in this review. Immunosuppression was administered per center protocol: antithymocyte globulin (ATG) induction was administered to three patients based on high immunologic-risk status (panel reactive antibody greater than 20% and/or African American race) while basiliximab induction was given to one patient with low-immune risk status. Each received triple maintenance immune suppression with tacrolimus, mycophenolate, and prednisone. All four patients were high serologic risk for CMV (donor IgG positive/recipient IgG negative) and were to receive valganciclovir 450 mg daily for 6 months with routine CMV polymerase chain reaction (PCR) screening at prespecified time points (1, 3, 6, 9, and 12 months) after transplant and when clinically warranted.

Treatment of CMV disease in clinically stable patients consisted of a valganciclovir 900 mg twice daily (adjusted for renal function) induction period for 21–28 days, followed by a 900 mg/day maintenance phase for an additional 28 days or until two consecutive negative PCRs (defined as <300 copies/mL) were achieved two weeks apart. Interestingly, all four patients included in this report developed “breakthrough” viremia in conjunction with symptoms during the period of prophylaxis. Therefore, valganciclovir dosages were increased to 900 mg twice daily (adjusted for renal impairment). Genotypic analysis for UL97 or UL54 mutations was performed upon failure to eradicate detectable viremia on treatment dosages of valganciclovir. All patients demonstrated confirmation of UL97 mutations and absence of UL54 mutations with documented resistance only to ganciclovir. Each patient was then initiated on combined therapy with intravenous (IV) cidofovir 1-2 mg per kg in 500–1000 mL of normal saline and CMV-IgG (Cytogam, CSL Behring AG, King of Prussia, PA) 100 mg per kg. Oral probenecid was administered at a dosage of 2 grams, 1 hour prior to and 4 hours after the completion of each cidofovir infusion. In addition, maintenance immunosuppressants were reduced to attain tacrolimus levels of 4–8 ng/mL and mycophenolate mofetil dosages of 500 mg twice daily. Combination therapy and PCRs were repeated every 2 weeks until two consecutive negative PCRs were achieved, at which point treatment was discontinued and PCR monitoring became less frequent.

## 3. Results

### 3.1. Patient Characteristics

Baseline characteristics are shown in Table 1. The patients were young, with a mean age of 33 years, and all were male. Three of four patients were African American and the same three received deceased donor kidneys. One patient (Patient 1) had an episode of acute rejection in the first posttransplant month requiring ATG treatment prior to CMV detection. This patient subsequently had the highest PCR detectable while on valganciclovir prophylaxis. The mean time from transplant to the first detectable CMV PCR was 114 days. Because “low-dose” valganciclovir in some patients may actually be appropriate dosing based on the presence of renal dysfunction, we evaluated renal function using both estimated 4-variable glomerular filtration rate (GFR) and Cockcroft-Gault equations. At the time of detectable CMV replication, only one patient (Patient 4) had a GFR greater than 60 mL/min/1.73 m^2^. However, 3 of 4 patients had a creatinine clearance greater than 60 mL/min using the Cockcroft-Gault method. Therefore, according to approved labeling of valganciclovir, three of the four patients were indeed receiving “low-dose” valganciclovir during breakthrough viremia detection.

### 3.2. GCV-R Diagnosis and Treatment

Viral and treatment characteristics are shown in Table 2. Genotypic assessment and diagnosis of GCV-R CMV were made at a mean of 103 days from the initial CMV PCR detection date, after persistence of detectable PCRs despite treatment dosages of valganciclovir. The average duration of valganciclovir exposure at the time of resistance diagnosis, including posttransplant prophylaxis, was therefore 217 days. All patients experienced CMV syndrome, with a predominance of fever, leukopenia, or malaise. In addition, Patient 1 had gastrointestinal symptoms, although a biopsy was not performed to evaluate for tissue invasion.

**Figure 1:**
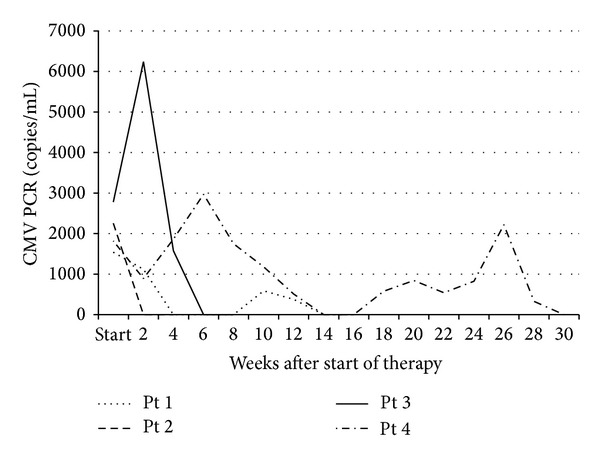
CMV PCR trend following initiation of therapy. Patients 1 and 4 developed recurrent viremia following discontinuation of therapy. Viral load became undetectable quickly in Patient 1 after resumption of therapy and after several dosages in Patient 4.

Each patient tolerated cidofovir and CMV-IgG infusions well without any reactions. Two consecutive negative PCRs were attained in all patients, occurring after an average of 5 treatments and a median of 42 (range 14 to 105) days from the date of the first infusion (Figure 1). Following discontinuation of therapy, Patients 1 and 4 each developed recurrent viremia. In each patient, the recurrence was not associated with symptoms; however, treatment was reinitiated. Viremia subsequently cleared again after 2 additional courses of cidofovir/CMV-IgG in Patient 1 and after 10 doses of cidofovir alone in Patient 4. In the latter patient, a repeat genotype was obtained, demonstrating the same UL97 mutation site and conferring resistance only to ganciclovir.

### 3.3. Graft Outcomes and Follow-Up

Renal function remained stable in all patients (Table 2). The mean change in GFR from the time of GCV-R diagnosis to the completion of therapy was −1 mL/min. At the time of this writing and after a mean follow-up of 21 months since the final dose of cidofovir, all patients are currently alive with functioning grafts. None of the patients developed recurrent disease and none experienced acute rejection throughout the follow-up period.

## 4. Discussion

Available data on the use of cidofovir for treatment of CMV in solid organ transplant recipients is scarce. While cidofovir is contraindicated in patients with reduced renal function, several reports describe its safety at lower dosages for the treatment of polyomavirus-induced nephropathy [9]. Furthermore, based on limited pharmacokinetic data in renally impaired patients, reduced dosages of cidofovir may provide adequate antiviral concentrations [10]. This led us to investigate the ability to use reduced-dose cidofovir to eliminate mild cases of GCV-R CMV disease. In addition, each patient received CMV-IgG for two reasons: first, to augment antiviral treatment by providing CMV antibodies in previously seronegative patients and, second, to take advantage of the potential immunomodulatory effects in an effort to prevent rejection [11]. In all four patients, this regimen led to resolution of GCV-R disease without deterioration of renal function or rejection.

Antiviral-resistant CMV has been reported in up to 14% of transplant recipients, with the highest rates occurring in high-risk serostatus recipients and lung transplant recipients [5, 12–14]. Ganciclovir, a guanosine analogue, requires three stages of phosphorylation in order to exert its inhibitory effect on CMV DNA polymerase. The initial phosphorylation occurs via viral UL97 phosphotransferase, which is followed by two subsequent phosphorylations by host cellular kinases. The final triphosphorylated form of ganciclovir preferentially inhibits viral UL54 DNA polymerase. Resistance can be conferred by mutations at the UL97 kinase and UL54 polymerase genes [15, 16]. Mutations in UL97 alone can result in resistance to ganciclovir and other nucleoside analogues by inhibition of the initial phosphorylation step. Mutations in UL54, albeit much less common, can confer resistance to cidofovir and foscarnet. Inadequate drug exposure and prolonged duration of antivirals have been attributed to the development of resistant CMV strains [17].

GCV-R CMV is a serious complication in solid organ transplant recipients. In addition to its significant morbidity and mortality, treatment options for GCV-R CMV are also associated with severe adverse outcomes. Recommended options such as cidofovir and foscarnet are associated with a high incidence of nephrotoxicity, often resulting in renal failure in both kidney and non-kidney transplant recipients [12, 13, 18, 19]. Additionally, rejection may also occur as a result of reduced immunosuppression and/or indirect effects associated with CMV infection [1].

While foscarnet is considered the preferred treatment for GCV-R, our own experience with the agent has been disappointing due to a very high incidence of acute renal failure requiring hemodialysis in patients with more severe disease. Given our familiarity in using cidofovir as a treatment option for polyomavirus nephropathy and the ability to use probenecid and hydration as nephroprotective measures, we chose this agent in these four cases of milder disease. Cidofovir is approved in the United States for CMV retinitis in patients with acquired immunodeficiency syndrome (AIDS) [8]. The drug requires phosphorylations to monophosphate and diphosphate (active) forms by pyrimidine nucleoside diphosphate kinase and nucleoside diphosphate kinase, respectively. The final metabolite, cidofovir diphosphate, inhibits CMV DNA polymerase, a product of gene UL54. While each of our patients demonstrated mutations only in UL97, development of resistance to cidofovir was an obvious concern. However, due to the relatively mild nature of disease and the presence of renal dysfunction in each patient, we felt that a reduced dose would be appropriate. A genotype was repeated in one patient with a slight rise in PCR after resuming cidofovir and was confirmed negative for the UL54 mutation. The patient subsequently cleared viremia after repeated dosing. An interesting finding in our cohort was that each patient developed their initial infection while on valganciclovir prophylaxis. This suggests that “breakthrough” CMV viremia during prophylaxis should warrant testing for antiviral resistance.

## 5. Conclusion

Treatment of GCV-R CMV in solid organ transplant recipients is challenged by dose-limiting toxicities of effective agents and potential graft injury due to indirect viral effects or rejection associated with lowering of immunosuppressive agents. In our experience, 4 consecutive patients with documented UL97 mutations conferring resistance to GCV were successfully treated with a regimen of reduced-dose cidofovir and CMV-IgG. It is important to highlight that all patients were diagnosed relatively early in the course of their infections, making this conservative approach more plausible. Undoubtedly, a more aggressive treatment would be required in patients with greater disease severity. We also cannot rule out the impact and importance of reduction of immunosuppression in the management of patients with GCV-R CMV. Nevertheless, all patients experienced viral eradication, and, importantly, none developed nephrotoxicity or rejection. We conclude that this regimen in addition to reduction of immunosuppression may serve as a treatment option in patients with mild GCV-R CMV disease.

## Figures and Tables

**Table 1:** Baseline characteristics of four D+/R− patients with confirmed ganciclovir-resistant CMV.

Patient number	Age/race/sex/weight (kg)	Donor type/PRA (%)	Induction	Time to 1st PCR (days)	1st PCR (copies)	SCr/GFR/CrCl at time of 1st PCR	Dose-adjusted for CrCl at time of 1st PCR (mg/day)	Peak PCR (copies)
1	28/AA/M/95	D/16	ATG	178	42807	2.2/43/53	900	42807
2	39/W/M/105	L/0	IL2-ra	90	1857	1.7/45/74	450	2257
3	32/AA/M/116	D/0	ATG	85	741	1.8/53/67	450	6240
4	32/AA/M/82	D/88	ATG	104	2987	1.5/65/73	450	2987

ATG: antithymocyte globulin; AA: African American; CrCl: creatinine clearance (mL/min); D: deceased donor; GFR: glomerular filtration rate (mL/min/1.73^2^); IL2-ra: interleukin 2 receptor antagonist; L: living donor; M: male; PCR: polymerase chain reaction; PRA: panel reactive antibodies; SCr: serum creatinine (mg/dL); W: White.

**Table 2:** Characteristics of ganciclovir-resistant cytomegalovirus and treatment.

Patient number	Time from 1st PCR to GCV-R diagnosis (days)	UL97 mutation site	PCR at time of GCV-R diagnosis (copies)	SCr/GFR/CrCl at time of GCV-R diagnosis	Total VGC exposure prior to GCV-R diagnosis (days)	CMV disease category	Total number of cidofovir infusions	GFR at end of treatment
1	82	A594V	1538	1.8/54/65	260	Syndrome, suspected GI invasion	7	45
2	162	A594V	2257	1.9/39/66	252	Syndrome	2	45
3	101	L595W	2777	1.6/61/73	186	Syndrome	5	61
4	65	L595S	1818	1.6/61/69	169	Syndrome	17	60

CrCl: creatinine clearance (mL/min); GCV-R: ganciclovir-resistant; GFR: glomerular filtration rate (mL/min/1.73^2^); GI: gastrointestinal; PCR: polymerase chain reaction; SCr: serum creatinine (mg/dL); VGC: valganciclovir.
